# Supplementary material for: Clinical efficacy and biomarker analysis of dual PD-1/CTLA-4 blockade in recurrent/metastatic EBV-associated nasopharyngeal carcinoma
Source: Nat Commun. 2023 May 15;14:2781. doi: 10.1038/s41467-023-38407-7 (PMC10184620; doi:10.1038/s41467-023-38407-7)
Supplement: Supplementary file 1 — Supplementary Information [file 41467_2023_38407_MOESM1_ESM.pdf]

Clinical efficacy and biomarker analysis of dual PD-1/CTLA-4 blockade in recurrent/metastatic EBV-associated nasopharyngeal carcinoma

Supplementary Information

Supplementary Table 1. Patient characteristics 2

Supplementary Table 2. Details on treatment-related adverse events 4

Supplementary Table 3. Prior treatment and EBV associations with depth of response 5

Supplementary Table 4. Samples for exploratory analysis. 6

Supplementary Table 5. Antibodies used for multiplex IHC/IF staining 7

Supplementary Figure 1 8

Supplementary Figure 2. 9

Supplementary Figure 3. 10

Supplementary Figure 4. 11

Supplementary References 13

Supplementary Table 1. Patient characteristics

	Number of patients (%) N=40
Date enrolled into study	21 Jul 2017 to 22 Aug 2019
Date of start of treatment for patients	31 Jul 2017 to 5 Sep 2019
Age at start of trial, years old	
Median (IQR)	53 (47.8, 61.1)
Range	23 to 73
Gender	
Male	33 (82.5)
Female	7 (17.5)
Ethnic group	
Chinese	36 (90.0)
Malay	3 (7.5)
Caucasian	1 (2.5)
T-stage	
T0	1 (2.5)
T1	3 (7.5)
T2	4 (10.0)
T3	9 (22.5)
T4	21 (52.5)
TX	2 (5.0)
N-stage	
N0	3 (7.5)
N1	8 (20.0)
N2	16 (40.0)
N3	7 (17.5)
N3B	4 (10.0)
NX	2 (5.0)
M-stage	
M0	22 (55.0)
M1	18 (45.0)
WHO Staging	
I	1 (2.5)
II	3 (7.5)
III	36 (90.0)
AJCC Staging	
2	2 (5.0)
3	7 (17.5)
4A	9 (22.5)
4B	7 (17.5)
4C	15 (37.5)
ECOG performance status at baseline	
0	12 (30.0)
1	28 (70.0)
Histopathologic type	
Squamous Cell, Non-keratinising	13 (32.5)
Undifferentiated Cell	24 (60.0)
Unknown	1 (2.5)
Not reported	2 (5.0)
Disease Status	
Metastatic	34 (85.0)
Recurrent	4 (10.0)
Not reported	2 (5.0)
Sites of metastatic disease (for patients with metastatic disease, n=34)	
Single site	20 (58.8)
Lung,	*5 (14.7)*

Bone,	*5 (14.7)*
Liver,	*5 (14.7)*
Distant Lymph Node	*2 (5.9)*
Neck	*1 (2.9)*
Oropharyngeal	*1 (2.9)*
Retropharyngeal Soft Tissue Bid Mediastinal Lymph	*1 (2.9)*
Two sites	11 (32.4)
Lung,Bone,	*3 (8.8)*
Lung,Liver,	*1 (2.9)*
Bone,Adrenal	*1 (2.9)*
Bone,Distant Lymph Node	*3 (8.8)*
Bone,Liver,	*1 (2.9)*
Liver,Distant Lymph Node	*2 (5.9)*
Three sites	3 (8.8)
Lung,Bone,Liver,	*1 (2.9)*
Bone,Liver,Distant Lymph Node	*2 (5.9)*
Site of metastatic disease (for patients with metastatic disease, n=34)	
Lung	10 (29.4)
Bone	16 (47.1)
Liver	12 (35.3)
Adrenal	1 (2.9)
Distant Lymph Node	9 (26.5)
Neck	1 (2.9)
Oropharyngeal	1 (2.9)
Retropharyngeal Soft Tissue Bid Mediastinal Lymph	1 (2.9)
Prior treatment for:	
Locally advanced disease only	2 (5.0)
Recurrent/metastatic disease only	17 (42.5)
LA and first-line R/M therapy	21 (52.5)
Prior treatment regimen for recurrent/metastatic disease (n=38)	
Carboplatin + TS - 1	1 (2.6)
Cisplatin + TS - 1	1 (2.6)
Cisplatin/Carboplatin + Fluorouracil	1 (2.6)
Endoxan	1 (2.6)
Gemcitabine + Carboplatin + T - Cell Infusion	1 (2.6)
Gemcitabine + Cisplatin/Carboplatin	28 (73.7)
Tegafur/Uracil	4 (10.5)
Xeloda	1 (2.6)
Prior treatment regimens for LA disease (n=23)	
Bevacizumab + Gemcitabine + Cisplatin	2 (8.7)
Carboplatin followed by (Cisplatin/Carboplatin + Fluorouracil)	1 (4.3)
Cisplatin	6 (26.1)
Cisplatin followed by (Cisplatin/Carboplatin + Fluorouracil)	2 (8.7)
Cisplatin followed by (Tegafur/Uracil)	1 (4.3)
Cisplatin/Carboplatin + Fluorouracil	4 (17.4)
Gemcitabine + Cisplatin/Carboplatin	1 (4.3)
Mitomycin + Epirubicin + Cisplatin + Fluorouracil + Leucovorin	4 (17.4)
Tegafur/Uracil followed by Cisplatin	1 (4.3)
Unknown	1 (4.3)
Number of patients who had prior RT	
No	12 (30.0)
Yes	26 (65.0)
Unknown	2 (5.0)

Supplementary Table 2. Details on treatment-related adverse events

	Number of patients with event (%)					
	**Grade 1**	**Grade 2**	**Grade 3**	**Grade 4**	**Grade 5**	**Any Grade**
Any trAE/SAE	14 (35)	13 (33)	4 (10)	2 (5)	1 (3)	34 (85)
Endocrine						
Adrenal insufficiency	0	1 (3)	2 (5)	0	0	3 (8)
Hyperthyroidism	0	1 (3)	0	0	0	1 (3)
Hypothyroidism	3 (8)	8 (20)	0	0	0	11 (28)
Gastrointestinal						
Constipation	1 (3)	0	0	0	0	1 (3)
Bloating	1 (3)	0	0	0	0	1 (3)
Diarrhea	3 (8)	0	0	0	0	3 (8)
Vomiting	1 (3)	0	0	0	0	1 (3)
Hematological						
Anemia	0	1 (3)	0	0	0	1 (3)
Neutropenia	0	1 (3)	0	0	0	1 (3)
Hepatobiliary						
ALT increase	4 (10)	1 (3)	0	0	0	5 (13)
AST increase	4 (10)	1 (3)	1 (3)	0	0	6 (15)
Lipase increase	0	0	0	2 (5)	0	2 (5)
Amylase increase	0	1 (3)	1 (3)	0	0	2 (5)
Bilirubin increase	1 (3)	0	0	0	0	1 (3)
Musculoskeletal						
Myalgia	1 (3)	0	0	0	0	1 (3)
Myositis	0	0	1 (3)	0	0	1 (3)
Neuromuscular junction disorder	0	0	1 (3)	0	0	1 (3)
Skin						
Dermatitis	2 (5)	0	0	0	0	2 (5)
Rash acneiform	3 (8)	0	0	0	0	3 (8)
Rash maculo-papular	11 (28)	5 (13)	0	0	0	16 (40)
Pruritus	2 (5)	0	0	0	0	2 (5)
Genitourinary						
Cystitis	0	0	1 (3)	0	0	1 (3)
Respiratory						
Cough	1 (3)	0	0	0	0	1 (3)
Pneumonia	0	0	1 (3)	0	0	1 (3)
Dyspnea	1 (3)	0	0	0	0	1 (3)
Rhinitis	1 (3)	0	0	0	0	1 (3)
Metabolic						
Hyperglycemia	0	1 (3)	1 (3)	0	0	2 (5)
Hyponatremia	0	1 (3)	0	0	0	1 (3)
Hypomagnesemia	1 (3)	0	0	0	0	1 (3)
General						
Anorexia	1 (3)	0	0	0	0	1 (3)
Fatigue	7 (18)	1 (3)	0	0	0	8 (20)
Fever	5 (13)	1 (3)	0	0	0	6 (15)
Pain	1 (3)	0	0	0	0	1 (3)
Edema	1 (3)	0	0	0	0	1 (3)
Others						
Adverse drug reaction (nivolumab)	0	1 (3)	0	0	0	1 (3)
Death	0	0	0	0	1 (3)	1 (3)

Supplementary Table 3. Prior treatment and EBV associations with depth of response

	n	BOR, n (%)	mPFS, , mths (95%	mOS, mths (95%	
**ECOG**					
0	12	6 (50.0%)	5.79 (1.68 11.70)	20.59 (2.17, 27.59)	
1	28	9 (32.1%)	5.29 (2.79, 8.38)	14.86 (10.36, NE)	
		p=0.3110^	p=0.8548	p=0.9094	
**Prior RT#**					
No	12	4 (33.3%)	4.03 (1.84, 6.41)	18.56 (7.17, 30.01)	
Yes	26	11 (42.3%)	5.88 (3.19 10.39)	21.71 (13.12, NE)	
		p=0.7281	p=0.1396	p=0.6146	
**Prior Gem***					
Yes	31	10 (32.3%)	5.26 (2.73, 6.28)	17.65 (10.19,	
No	8	5 (62.5%)	11.83 (1.74, NE)	NR	
		p=0.2202^	p=0.0628	p=0.0744	
**Prior Cisplatin***					
Yes	31	10 (32.3%)	5.26 (2.76, 6.28)	21.71 (10.36,	*OS results to be*
No	8	5 (62.5%)	10.39 (2.53, 17.65)	19.84 (7.00, NE)	*interpreted with care*
		p=0.2202^	p=0.2456	p=0.9954	*due to small sample*

Excluded two patients with no Prior RT information
* Excluded one patient with unknown prior treatment regimen
^ p-value calculated using Fisher Exact test

	No. of CR/PR (RECIST)	Median DoR, mths (95% CI)	No. of patients with tumour shrinkage	Depth of Response, % Median (IQR)
**EBV DNA groups**				
EBV<7800 IU/ml	9	5.85 (3.85, 8.98)	11	50.22 (33.33, 68.32)
EBV≥7800 IU/ml	0	-	4	18.43 (13.23, 35.91)
				Mann Whitney U p=0.1027

	Depth of Response, %			
	All patients (n=26)		Cohort of patient with tumour shrinkage	
**EBV DNA groups**	No. of	Median (IQR)	No. of pts w tumour	Median (IQR)
EBV<7800 IU/ml	15	36.86 (-13.91, 58.22)	11	50.22 (33.33, 68.32)
EBV≥7800 IU/ml	10#	-70.59 (-125.51,	4	18.43 (13.23, 35.91)
		Mann Whitney U p=0.0060		Mann Whitney U p=0.1027

1 patient excluded as patient passed away before first planned post-treatment tumor scan

	Treatment duration, weeks	
	No. of patients	Median (IQR)
**EBV DNA groups**		
EBV<7800 IU/ml	15	23.93 (9.97, 45.87)
EBV≥7800 IU/ml	11	9.97 (3.99, 21.80)
		Mann Whitney U p=0.0515

Supplementary Table 4. Samples for exploratory analysis.

Patient ID	Site of Biopsy	RECIST Response	Site Response	Paired On-Treatment Biospy	Nanostring IO360*	Whole Exome-Seq^	Biopsy FFPE (mIHC/IF)^	Included in Gene Signature analysis
1001	Primary	PR	PR	Yes	Yes	Yes	Yes	Yes
1002	Primary	SD	SD	Yes	Yes	Yes	Yes	No
1003	Primary	PD	PD	Yes	Yes	Yes	Yes	Yes
1004	Primary	PR	PR	Yes	Yes	Yes	Yes	Yes
1005	Liver	PD	PD	No	Yes	Yes	No	No
1007	Primary	PD	PD	Yes	Yes	Yes	Yes	Yes
1008	Primary	SD	SD	Yes	Yes	Yes	Yes	No
1010	Lymph Node	PD	PR	Yes	Yes	Yes	Yes	Yes
1012	Lymph Node	PR	PR	Yes	Yes	Yes	Yes	Yes
1013	Primary	PR	PR	Yes	Yes	Yes	Yes	Yes
1014	Lymph Node	PR	PR	Yes	Yes	No	Yes	Yes
1016	Lymph Node	PR	PR	No	Yes	Yes	No	No
1018	Lymph Node	SD	SD	Yes	Yes	Yes	Yes	No
1019	Primary	PD	PD	Yes	Yes	Yes	Yes	Yes
1020	Primary	PD	PD	Yes	Yes	Yes	Yes	Yes
1021	Primary	SD	SD	Yes	Yes	Yes	Yes	No
1023	Primary	PD	PD	No	Yes	Yes	No	No
1028	Primary	PD	PD	Yes	Yes	No	No	Yes
1029	Primary	PD	PR	Yes	Yes	Yes	Yes	Yes
1032	Primary	SD	SD	Yes	Yes	Yes	Yes	No
1033	Primary	SD	SD	Yes	Yes	Yes	Yes	No
1040	Primary	PR	PR	Yes	Yes	Yes	No	Yes

* Experiment and analysis ran for both pre- and on-treatment samples where applicable.

^ Experiment and analysis ran for pre-treatment samples only.

Supplementary Table 5. Antibodies used for multiplex IHC/IF staining

Antibody	Clone	Dilution	Catalogue No.	Source
CD39	OTI2B10	1:800	TA804559	Origene
CD8	4B11	1:100	NCL-L-CD8-4B11	Leica
FOXP3	136A/E7	1:200	ab20034	Abcam
TCF1	C63D9	1:600	2203S	CST
PD1	NAT105	1:200	315M-96	Cell Marque
CTLA4	IHC004	1:400	IHC004-100	GENEAB

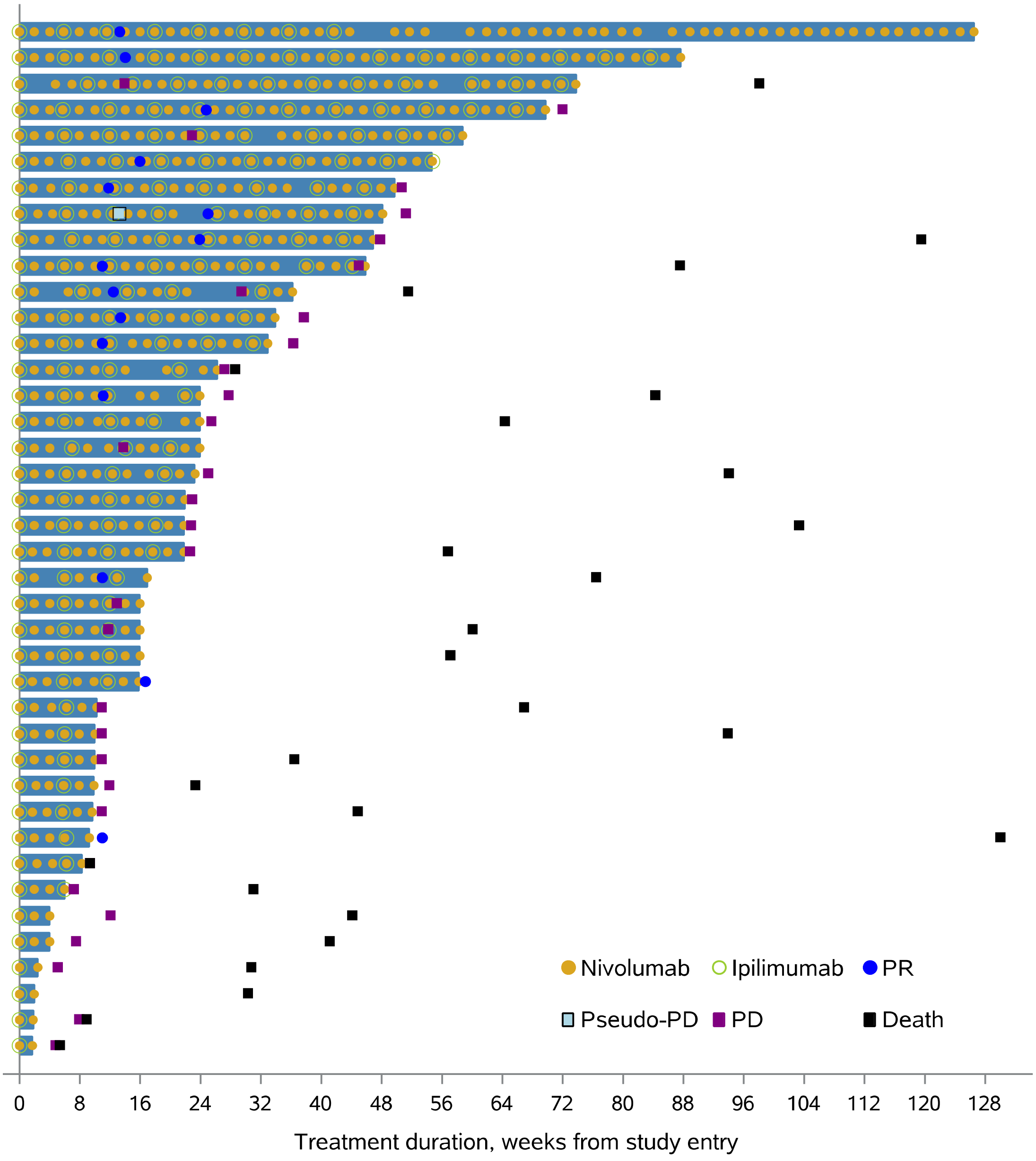

Supplementary Figure 1. Swimmers plot summarizing treatment duration, response, and outcomes over time for patients in study. n= 40.

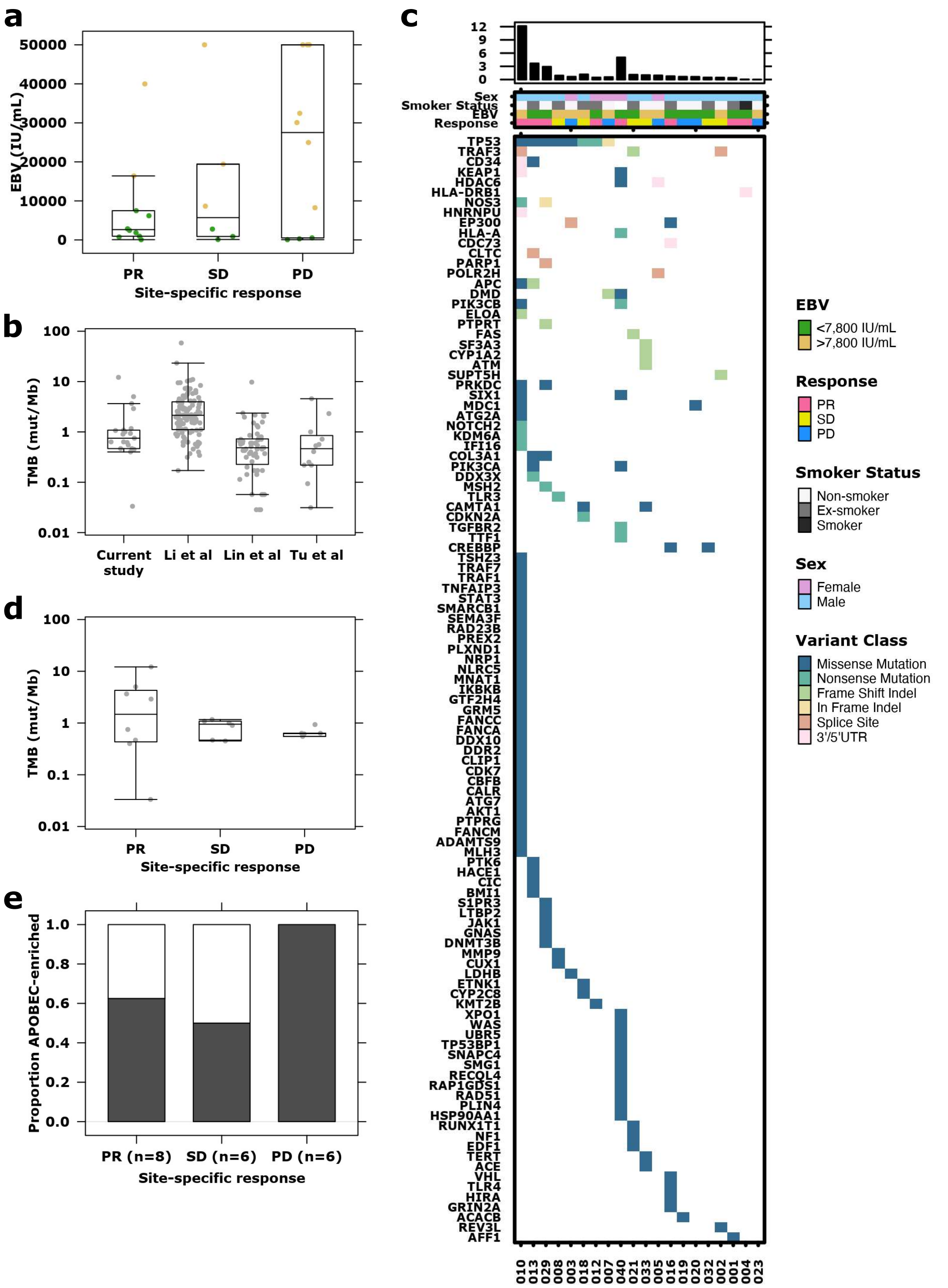

Supplementary Figure 2. Genomic profiles revealed by whole exome sequencing.

a. EBV viral load by site-specific response, with green and yellow showing EBV-low (<7,800 IU/mL) and EBV-high (>7,800 IU/mL) groups respectively: n_{PR} = 10, n_{SD} = 6, n_{PD}=10.
b. Comparison of tumor mutation burden across NPC genomics studies[1,2,3]: $n_{current\_study}$ = 21, n_{Li} [1] = 110, n_{Lin} [2] = 56, n_{Tu} [3] = 12.
c. Extended tumor co-mutation plot with tumor mutation burden and clinical covariates showing all mutated NPC-related genes in this cohort.
d. Tumor mutation burden by site-specific response: n_{PR} = 8, n_{SD} = 6, n_{PD} = 6.
e. Proportion of APOBEC-enriched samples by site-specific response.

(a,b,d) Tukey boxplots are shown with the box indicating quartiles with median at middle, and the whiskers drawn at the lowest and highest points within 1.5 interquartile range of the lower and upper quartiles, respectively.

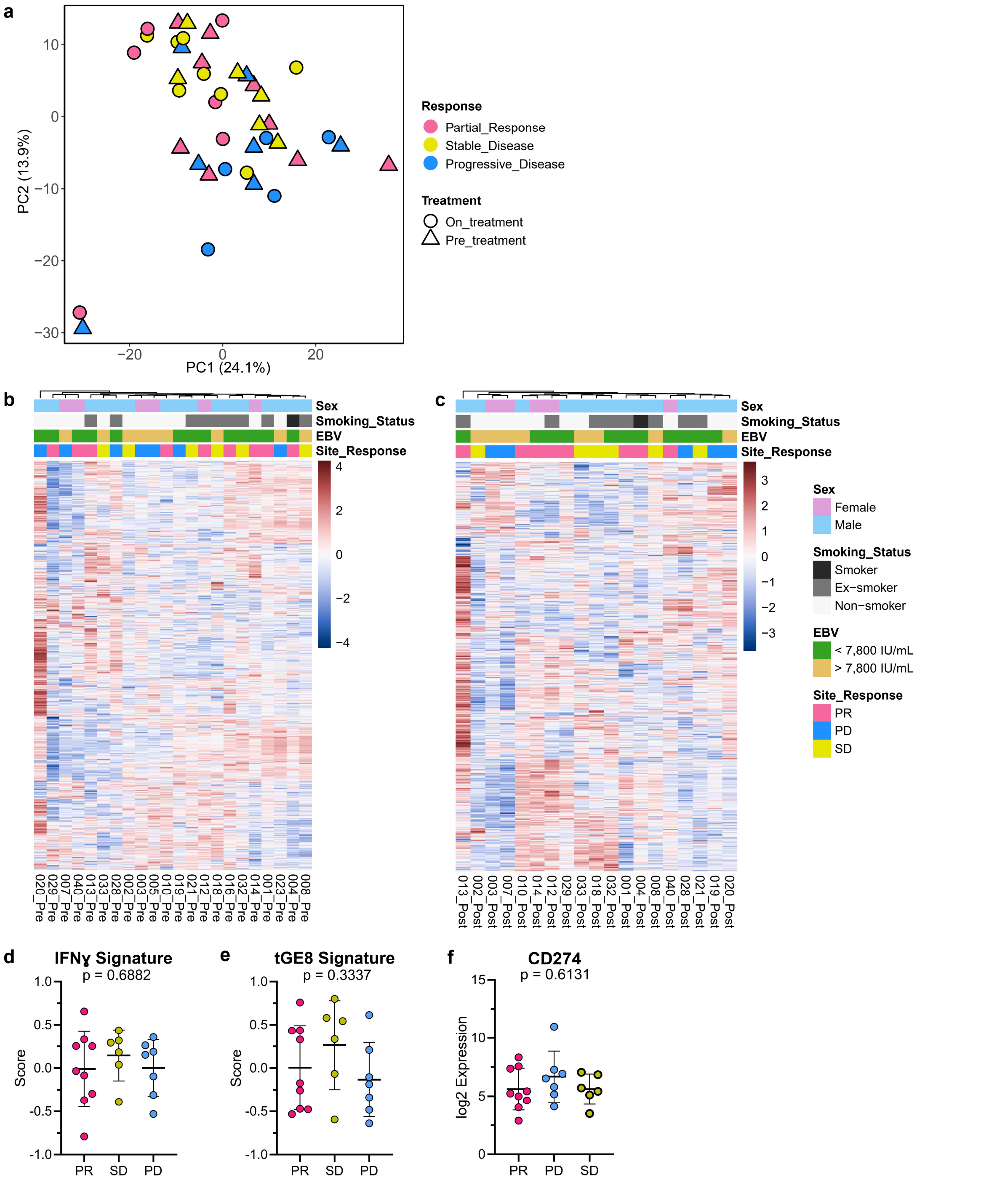

Supplementary Figure 3. Overview of gene expression profile using Nanostring IO360 panel.
a. PCA plot showing distribution of all samples used in the analysis (n= 41).
b-c. Heatmap showing unsupervised clustering of pre-treatment (n= 22) (a) and on-treatment (n= 19) (b) samples (750 genes).
d-f. Correlation between IFNɣ signature score[4] (d), cytotoxic T-cells transcriptional signature score[5] (e) and *CD274* (PD-L1) mRNA (f) expression with response at pre-treatment setting ($n_{PR} = 9$, $n_{SD} = 6$, $n_{PD} = 7$). Statistical analyses were performed with two-sided Kruskal-Wallis test. Line represents mean with SD. Each dot represents an individual sample.

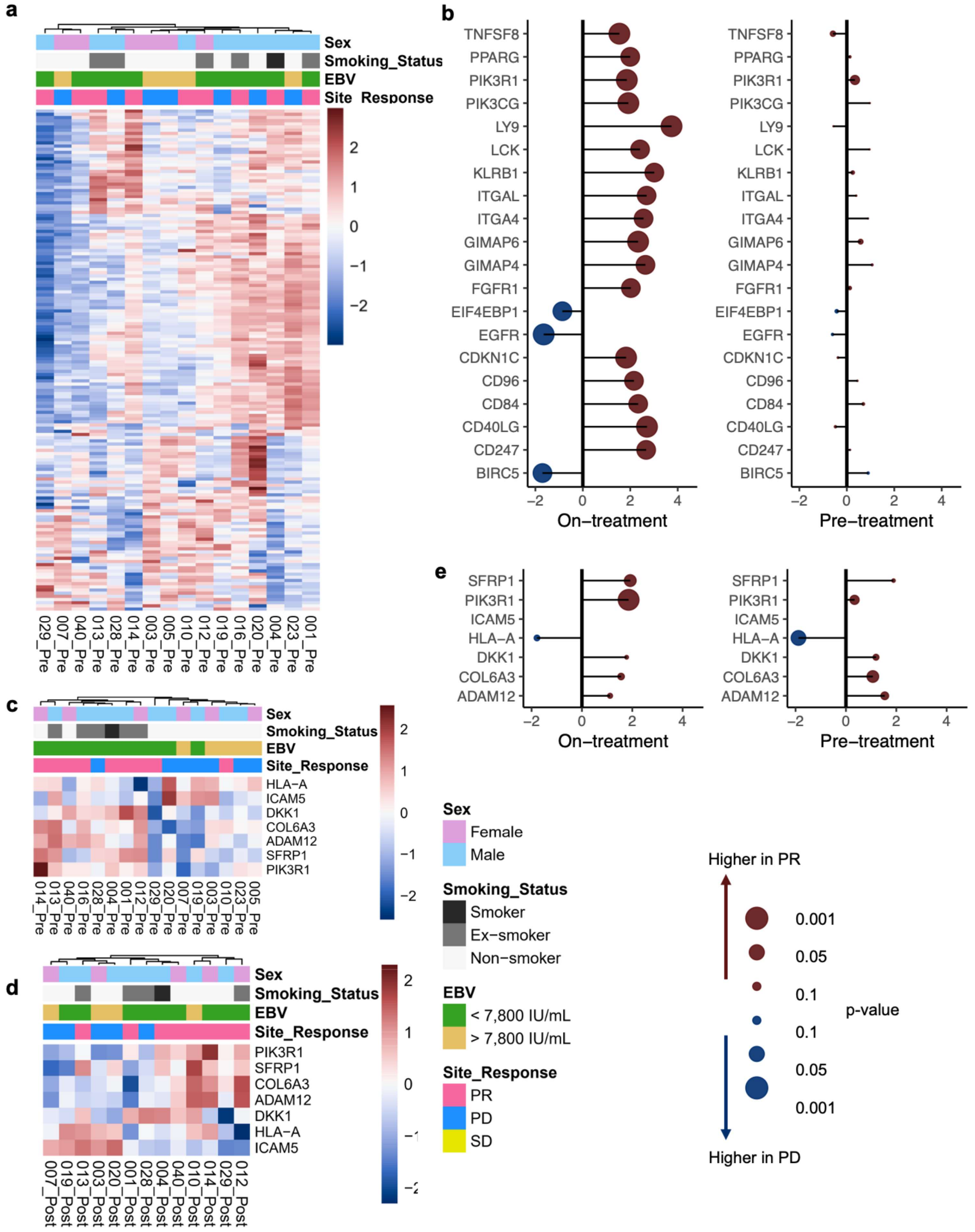

Supplementary Figure 4. Dynamics of treatment response in pre- *vs* on-treatment samples.
a. On-treatment genes of interest in pre-treatment samples.
b. Comparison of on-treatment genes of interest between PR (n=9) and PD (n=7) responders in on-treatment (left) and pre-treatment (right) samples. The length of the stem indicates the effect size of median difference in expression with points towards the left (blue dots) showing higher expression in PD and right (red dots) showing higher expression in PR. The size of the dot indicates P-values by Mann-Whitney U-test.
c-d. Pre-treatment genes of interest in (c) pre-treatment and (d) on-treatment samples.
e. Comparison of pre-treatment genes of interest between PR (n=8) and PD (n=6) responders in on-treatment (left) and pre-treatment (right) samples.

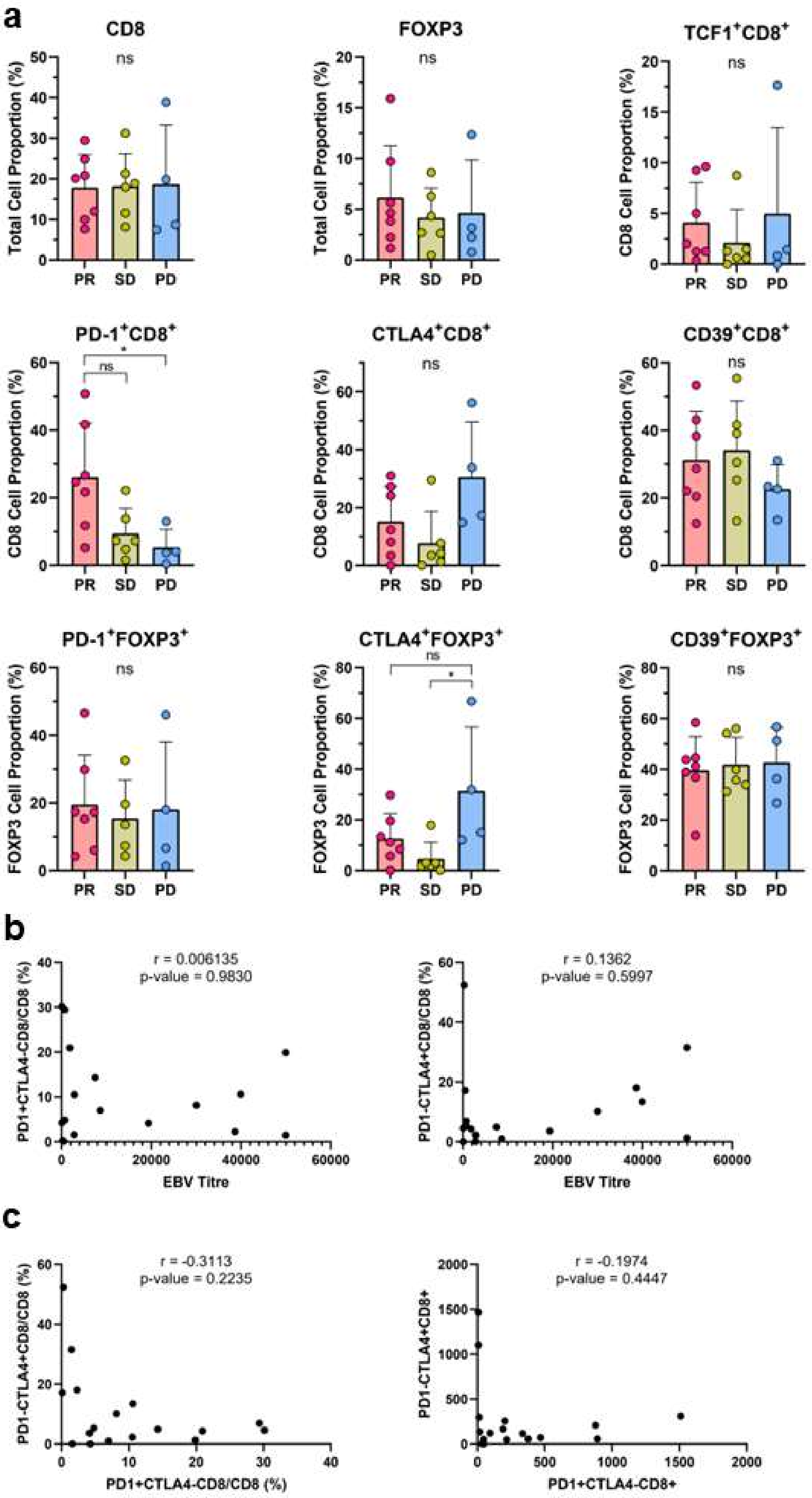

Supplementary Figure 5. Cell proportion quantification using multiplex-IHC/IF.

a. Cell type proportion with categorized by biopsy site response. $n_{PR} = 7$, $n_{SD} = 6$, $n_{PD} = 4$. Statistical analyses were performed using ANOVA with post-hoc Tukey test, adjusted for multiple comparison. Bar plot shows mean with SD.

b. Correlation plot between EBV titre and PD1$^+$CTLA4$^-$CD8 (left) or PD1$^-$CTLA4$^+$CD8 (right) T-cell proportion determined using mIHC/IF (Spearman correlation).

c. Correlation plot between PD1$^+$CTLA4$^-$CD8 and PD1$^-$CTLA4$^+$CD8 T-cell proportion (left) and absolute cell count (right).

(a, b, c) n = 17. Each dot represents an individual sample. Source data are provided as a Source Data file. PR: Partial response, SD: Stable disease, PD: Progressive disease.

Supplementary References

1. Li, Y.Y., et al. Exome and genome sequencing of nasopharynx cancer identifies NF-kappaB pathway activating mutations. Nat Commun 8, 14121 (2017).
2. Lin, D.C., et al. The genomic landscape of nasopharyngeal carcinoma. Nat Genet 46, 866-871 (2014).
3. Tu, C.*, et al.* Identification of genomic alterations in nasopharyngeal carcinoma and nasopharyngeal carcinoma-derived Epstein-Barr virus by whole-genome sequencing. *Carcinogenesis* **39**, 1517-1528 (2018).
4. Mariathasan, S., et al. TGFβ attenuates tumour response to PD-L1 blockade by contributing to exclusion of T cells. Nature 554, 544-548 (2018).
5. Ayers, M.*, et al.* IFN-γ–related mRNA profile predicts clinical response to PD-1 blockade. *The Journal of Clinical Investigation* **127**, 2930-2940 (2017).
