# Supplementary material for: Clinical efficacy and biomarker analysis of dual PD-1/CTLA-4 blockade in recurrent/metastatic EBV-associated nasopharyngeal carcinoma
Source: Nat Commun. 2023 May 15;14:2781. doi: 10.1038/s41467-023-38407-7 (PMC10184620; doi:10.1038/s41467-023-38407-7)
Supplement: Supplementary file 3 — Description of Additional Supplementary Files [file 41467_2023_38407_MOESM3_ESM.pdf]

Description of Additional Supplementary Files

File Name: Supplementary Data 1
Description: Differentially expressed genes between PD vs PR at on-treatment setting. Data underlying Figure 4a-b. Differential expression analysis comparing PR vs PD was iteratively performed using DESeq2 R package (v1.34.0) excluding one sample at a time. Overlapping genes were considered as a final and robust set of differentially expressed genes. P-value is obtained using Wald test, cutoff <0.05

File Name: Supplementary Data 2
Description: Pearson correlation of genes with time to progression at on-treatment setting. Data underlying Figure 4d. R and two-sided p values determined using Pearson correlation statistical analysis.

File Name: Supplementary Data 3
Description: Comparison of all (pre- and on-treatment) genes of interest in each condition (pre- vs. on-treatment). Data underlying Supplementary Figures 4b and 4e. P-value is obtained using Wald test, cutoff <0.05.

File Name: Supplementary Data 4
Description: Differentially expressed genes comparing pre-treatment PR vs PD and on-treatment PR vs PD. Data underlying Figure 4e. P value obtained using Wald test, cutoff <0.05.
