# Supplementary material for: Clinical efficacy and biomarker analysis of dual PD-1/CTLA-4 blockade in recurrent/metastatic EBV-associated nasopharyngeal carcinoma
Source: Nat Commun. 2023 May 15;14:2781. doi: 10.1038/s41467-023-38407-7 (PMC10184620; doi:10.1038/s41467-023-38407-7)
Supplement: Supplementary file 8 — Reporting Summary [file 41467_2023_38407_MOESM8_ESM.pdf]

nature portfolio

Corresponding author(s): N Gopalakrishna Iyer

Last updated by author(s): Apr 14, 2023

Reporting Summary

Nature Portfolio wishes to improve the reproducibility of the work that we publish. This form provides structure for consistency and transparency in reporting. For further information on Nature Portfolio policies, see our Editorial Policies and the Editorial Policy Checklist.

Statistics

For all statistical analyses, confirm that the following items are present in the figure legend, table legend, main text, or Methods section.

n/a	Confirmed	
☐	☒	The exact sample size (*n*) for each experimental group/condition, given as a discrete number and unit of measurement
☐	☒	A statement on whether measurements were taken from distinct samples or whether the same sample was measured repeatedly
☐	☒	The statistical test(s) used AND whether they are one- or two-sided<br>*Only common tests should be described solely by name; describe more complex techniques in the Methods section.*
☐	☒	A description of all covariates tested
☐	☒	A description of any assumptions or corrections, such as tests of normality and adjustment for multiple comparisons
☐	☒	A full description of the statistical parameters including central tendency (e.g. means) or other basic estimates (e.g. regression coefficient) AND variation (e.g. standard deviation) or associated estimates of uncertainty (e.g. confidence intervals)
☐	☒	For null hypothesis testing, the test statistic (e.g. *F*, *t*, *r*) with confidence intervals, effect sizes, degrees of freedom and *P* value noted<br>*Give P values as exact values whenever suitable.*
☒	☐	For Bayesian analysis, information on the choice of priors and Markov chain Monte Carlo settings
☒	☐	For hierarchical and complex designs, identification of the appropriate level for tests and full reporting of outcomes
☐	☒	Estimates of effect sizes (e.g. Cohen's *d*, Pearson's *r*), indicating how they were calculated

Our web collection on statistics for biologists contains articles on many of the points above.

Software and code

Policy information about availability of computer code

Data collection	No software was used for data collection
Data analysis	nSolver analysis software v4.0 (NanoString Technologies, Inc.), inform software v2.4.6 (Akoya Biosciences), SAS v9.4 (SAS Institute Inc., Cary, NC), GraphPad Prism v8.0.0(GraphPad Software, Inc., San Diego, CA, USA), and R version 4.1 with packages maftools (v2.8.05), survival (v3.2-13), survminer (v0.4.9), pROC (v1.18.0), BoutrosLab.plotting.general (v6.0.3), pheatmap (v1.0.12, and ggplot2 (v3.3.5), GRCh38 using BWA mem (v0.7.17), samtools (v1.7), GATK (v4.1.8.0), Strelka2 (v2.9.10), Lancet (v1.1.0), deconstructSigs (v1.8.0), RUVSeq (v1.24.0), clusterProfiler (v4.1.1).

For manuscripts utilizing custom algorithms or software that are central to the research but not yet described in published literature, software must be made available to editors and reviewers. We strongly encourage code deposition in a community repository (e.g. GitHub). See the Nature Portfolio guidelines for submitting code & software for further information.

Data

Policy information about availability of data

All manuscripts must include a data availability statement. This statement should provide the following information, where applicable:

- Accession codes, unique identifiers, or web links for publicly available datasets
- A description of any restrictions on data availability
- For clinical datasets or third party data, please ensure that the statement adheres to our policy

Data availability is subject to local rules and regulations. Patient data from a clinical trial is subject to patient confidentiality. Subjects did not provide consent for

their DNA or clinical data to be made publicly available. However, every reasonable effort will be made for scientifically valid requests. Requests for data should be made to the corresponding authors together with a detailed study plan and a commitment not to use the data and its derivatives for commercial purposes. The proposal will require approvals by the respective institutional review boards and the principal investigators. Requesting researchers will be required to sign a data access agreement with the relevant parties. The full study protocol and statistical analysis plan is available on reasonable request from collaborators. Given the restrictions posed by patient consent and institutional review boards, the raw WES data has not been deposited in a public repository but processed data can be made upon reasonable request. The raw Nanostring data is available in the GEO database under accession 559code GSE224450 [https://www.ncbi.nlm.nih.gov/geo/query/acc.cgi?acc=GSE224450]. The study synopsis is available in the Article, Supplementary Information, or Source data file.

Human research participants

Policy information about studies involving human research participants and Sex and Gender in Research.

Reporting on sex and gender	No gender specific analyses were performed because it was inconsequential in this study. The trial recruited male and female patients without any prejudice. Most patients in this trial were male (n=33/40; 82.5%) since NPC is a male predominated disease. Both females and males were used in our dataset without any prejudice.
Population characteristics	They were eligible if they had recurrent/metastatic undifferentiated NPC with detectable plasma EBV DNA at study entry. They had to be at least 20 years of age at study entry and of good ECOG PS 0/1. They had to have measurable disease per RECIST v1.1 criteria. They could not have received more than one line of prior palliative chemotherapy. Patients who progressed/relapsed within 6 months of definitive chemoradiation for locally advanced disease were considered to have chemotherapy-resistant disease, and patients who were not fit for platinum-based therapy disease were eligible.
Recruitment	Study population who met eligibility criteria were recruited from outpatient oncology clinics in 3 comprehensive cancer centres
Ethics oversight	The study was designed and conducted in compliance with ICH Good Clinical Practice guidelines and ethical principles described in the Declaration of Helsinki, regarding the use of human subjects in clinical trials. The study was approved by the respective Institutional Review Boards (Singapore Health Services Institutional Review Board, National Healthcare Group Domain Specific Review Board, and National Taiwan University Hospital Research Ethnics Committee)

Note that full information on the approval of the study protocol must also be provided in the manuscript.

Field-specific reporting

Please select the one below that is the best fit for your research. If you are not sure, read the appropriate sections before making your selection.

[x] Life sciences [ ] Behavioural & social sciences [ ] Ecological, evolutionary & environmental sciences

For a reference copy of the document with all sections, see nature.com/documents/nr-reporting-summary-flat.pdf

Life sciences study design

All studies must disclose on these points even when the disclosure is negative.

Sample size	The sample size for this single-arm Phase II trial was estimated using the Simon Two-Stage Minimax Design. Based on a historical control best overall response (BOR) rate of 25%, this trial aimed to investigate if the BOR rate of Nivolumab in combination with Ipilimumab was at least 45%. With power of 80% and one-sided type I error of 10%, a sample size of 26 patients was required. An expansion cohort of up to 14 patients was included to obtain additional safety/toxicity signals, and to increase the power and the precision of PFS/OS estimates for a subsequent Phase III trial. Hence, the trial planned to enroll a total of 40 patients.
Data exclusions	No data was excluded from reporting.
Replication	Not applicable in clinical trial setting.
Randomization	Non-randomized single arm clinical trial.
Blinding	Open label single arm study where blinding is not relevant.

Reporting for specific materials, systems and methods

We require information from authors about some types of materials, experimental systems and methods used in many studies. Here, indicate whether each material, system or method listed is relevant to your study. If you are not sure if a list item applies to your research, read the appropriate section before selecting a response.

Materials & experimental systems

n/a	Involved in the study	
☐	☒	Antibodies
☒	☐	Eukaryotic cell lines
☒	☐	Palaeontology and archaeology
☒	☐	Animals and other organisms
☐	☒	Clinical data
☒	☐	Dual use research of concern

Methods

n/a	Involved in the study	
☒	☐	ChIP-seq
☒	☐	Flow cytometry
☒	☐	MRI-based neuroimaging

Antibodies

Antibodies used	Primary antibodies: CD39 (Clone: OTI2B10; Cat no: TA804559, Origene), CD8 (Clone: 4B11; Cat no: NCL-L-CD8-4B11, Leica), FOXP3 (Clone: 136A/E7; Cat no: ab20034, Abcam), TCF1 (Clone: C63D9; Cat no: 2203S, CST), CTLA4 (Clone: IHC004; Cat no: IHC004-100, GENEAB), PD1 (Clone: NAT105; Cat no: 315M-96, Cell Marque); Secondary antibodies: BOND Polymer Refine Detection Kit (Catalog No: DS9800)
Validation	All antibodies were validated by the manufacturer, and antibody-specific staining was compared to isotype control when necessary by the manufacturer.<br><br>CD39 (https://www.origene.com/catalog/antibodies/primary-antibodies/ta804559/cd39-entpd1-mouse-monoclonal-antibody-clone-id-oti2b10) Reference: Yeong et al. JTO 2021, DOI:https://doi.org/10.1016/j.jtho.2021.04.016<br>CD8 (https://shop.leicabiosystems.com/en-sg/ihc-ish/ihc-primary-antibodies/pid-cd8) Reference: Yeong et al. JTO 2021, DOI:https://doi.org/10.1016/j.jtho.2021.04.016<br>FOXP3 (https://www.abcam.com/foxp3-antibody-236ae7-ab20034.html) Reference: Schutt et al. Oncotarget 2021, DOI: 10.18632/oncotarget.27907<br>TCF1 (https://www.cellsignal.com/products/primary-antibodies/tcf1-tcf7-c63d9-rabbit-mab/2203) Reference: Ding et al. Nat Com 2019, DOI: 10.1038/s41467-019-12125-5<br>CTLA4 (https://www.histoline.com/en/ihc004-100)<br>PD1 (https://www.cellmarque.com/antibodies/CM/2009/PD-1_NAT105) Reference: Thibult et al Int Immunol 2013, DOI: 10.1093/intimm/dxs098<br>Secondary Antibody (https://shop.leicabiosystems.com/en-sg/ihc-ish/detection-systems/pid-bond-polymer-refine-detection) Parra et al. Sci Rep 11, 4530 (2021). https://doi.org/10.1038/s41598-021-83858-x

Clinical data

Policy information about clinical studies

All manuscripts should comply with the ICMJEguidelines for publication of clinical research and a completedCONSORT checklist must be included with all submissions.

Clinical trial registration	clinicaltrials.gov NCT03097939
Study protocol	https://clinicaltrials.gov/ct2/show/NCT03097939
Data collection	Patients in this study analysis cohort were recruited from July 2017 to August 2019. Recruitment Site: National Cancer Centre Singapore, National University Hospital, Singapore, and National Taiwan University Hospital, Taiwan.
Outcomes	Primary Outcome: Best Overall Response Rate (BOR) by RECIST [Time Frame: From the start of treatment until disease progression/recurrence, up to 2 years]<br>Secondary Outcomes: Progression-free survival [Time Frame: Time from first dose with IO agents until objective tumour progression, or death from any cause, whichever occurs first, up to 2 years], Clinical benefit rate (CR/PR/SD), duration of response, overall survival, time to progression, and frequency of adverse events.
